# Beneficial Role of Phytochemicals on Oxidative Stress and Age-Related Diseases

**DOI:** 10.1155/2019/8748253

**Published:** 2019-04-07

**Authors:** Cinzia Forni, Francesco Facchiano, Manuela Bartoli, Stefano Pieretti, Antonio Facchiano, Daniela D'Arcangelo, Sandro Norelli, Giorgia Valle, Roberto Nisini, Simone Beninati, Claudio Tabolacci, Ravirajsinh N. Jadeja

**Affiliations:** ^1^Department of Biology, University of Rome “Tor Vergata”, Rome, Italy; ^2^Department of Oncology and Molecular Medicine, Istituto Superiore di Sanità, Rome, Italy; ^3^Department of Ophthalmology, Medical College of Georgia at Augusta University, Augusta, GA, USA; ^4^National Center for Drug Research and Evaluation, Istituto Superiore di Sanità, Rome, Italy; ^5^Laboratory of Molecular Oncology, Istituto Dermopatico dell'Immacolata, IDI-IRCCS, Rome, Italy; ^6^Department of Infectious Diseases, Istituto Superiore di Sanità, Rome, Italy; ^7^Department of Medicine, University Campus Bio-Medico, Rome, Italy; ^8^Department of Biochemistry and Molecular Biology, Medical College of Georgia at Augusta University, Augusta, GA, USA

## Abstract

Aging is related to a number of functional and morphological changes leading to progressive decline of the biological functions of an organism. Reactive Oxygen Species (ROS), released by several endogenous and exogenous processes, may cause important oxidative damage to DNA, proteins, and lipids, leading to important cellular dysfunctions. The imbalance between ROS production and antioxidant defenses brings to oxidative stress conditions and, related to accumulation of ROS, aging-associated diseases. The purpose of this review is to provide an overview of the most relevant data reported in literature on the natural compounds, mainly phytochemicals, with antioxidant activity and their potential protective effects on age-related diseases such as metabolic syndrome, diabetes, cardiovascular disease, cancer, neurodegenerative disease, and chronic inflammation, and possibly lower side effects, when compared to other drugs.

## 1. Introduction

**Figure 1:**
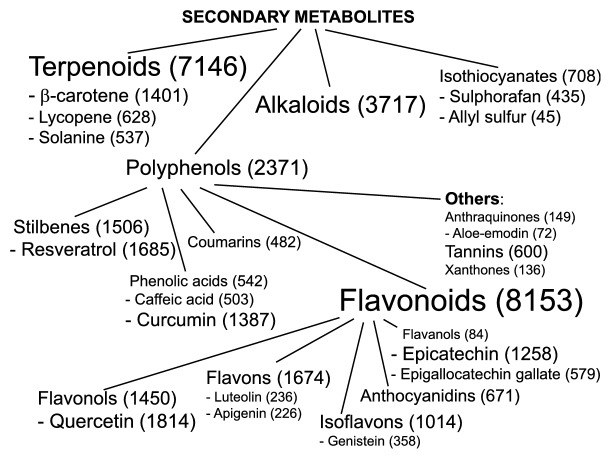
A word-tree-cloud showing the body of published evidence indexed on PubMed up today, regarding the most studied phytochemicals as related to oxidative stress. Between brackets, the number of published manuscripts containing the name of each phytochemical and “oxidative stress”, within Title or Abstract, is reported.

**Figure 2:**
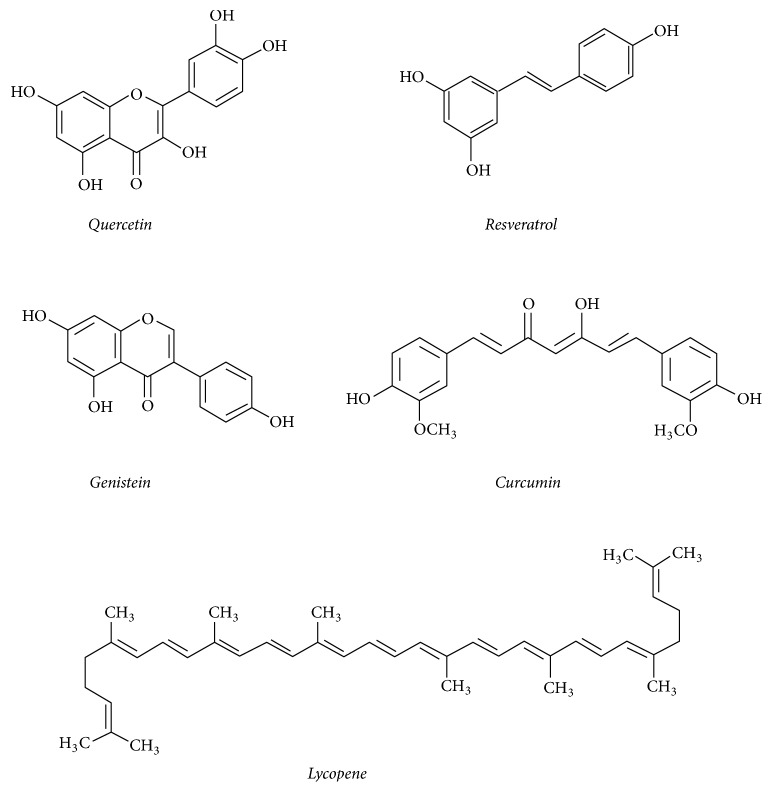
Structures of main secondary plant metabolites with demonstrated antioxidant and anti-inflammatory activity summarized in this review.

Aging in multicellular organisms is a biological process characterized by the progressive decline of cellular functions and by diminished tissue renewal capability, leading to a reduced ability to counteract the environmental stressors. Aging is controlled by several heterogeneous mechanisms involving genetic, epigenetic, and environmental factors [1, 2]. As largely demonstrated by literature data, aging has a strong relationship with several pathological conditions, including metabolic syndrome, obesity, cardiovascular diseases (CVD), cancer, and neurodegenerative diseases [3, 4]. Interestingly, the age-related pathologies are tightly associated with an increase in Reactive Oxygen Species (ROS) and subsequent oxidative stress [4]. In particular, a large number of studies emphasizes the antioxidant potential of natural compounds (“phytochemicals”) (Figure 1). Since oxidative stress and inflammation are closely related to pathophysiological processes, here we focused our attention on phytochemicals with well-known anti-inflammatory effects. Starting from the large available literature, the most used compounds and with the strongest evidence-based efficacy were summarized in Figure 2.

## 2. Plant Secondary Metabolites as Powerful Antioxidant Agents

Phytochemicals are a powerful group of compounds, belonging to secondary metabolites of plants and including a diverse range of chemical entities such as polyphenols, flavonoids, steroidal saponins, organosulphur compounds, and vitamins. They have important roles in plant development, being part of relevant physiological process, i.e., reproduction, symbiotic association, and interactions with other organisms and the environment. Even though most of these compounds occur constitutively, their synthesis can be enhanced under stress conditions, in a manner dependent on the growth conditions and on stressor [16–18]. In plants, following the exposure to stressful conditions, an oxidative burst may cause an imbalance between ROS production and scavenging, leading to the activation of reactive antioxidant enzymatic and non-enzymatic responses [16]. The first one includes changes in the activity of antioxidant enzymes, such as superoxide dismutase, peroxidases, and catalase, while the non-enzymatic response is related to the synthesis of low molecular (ascorbic acid, glutathione, carotenoids, phenolic acids, flavonoids, and others) and high molecular weight antioxidants (tannins).

Many of these plant metabolites have been tested on animal and human cells, showing very interesting biological activities (Table 1). They have been shown to be useful in pharmaceutical applications and in cosmetics, nutrition, and dietary supplements [19]. Plants have been always considered as source of food and medical compounds [20]: actually, up to 200 species are considered as medicinal plants and about 25% of the medicines have plants origins [21]. Most of phytochemicals, components of food, beverages, and herbal products are often reported in literature as “nutraceutical”, emphasizing their health promoting properties, including the prevention and treatment of pathologies like cancer, cardiovascular diseases, neural disorders, and Alzheimer's disease [22], as reported in the following paragraphs. Thus a plethora of information on the effects of phytochemicals on* in vitro* and* in vivo* systems is available in the literature. It is worthy to underline that the antioxidant activity of plant metabolites, detected by* in vitro* assays, does not always correspond to an effective action* in vivo.* This may be due to different metabolic processes that can affect their antioxidant activity [23] (see also paragraph 3 of this paper); thus some* in vitro* data have to be considered with caution.

The first antioxidant molecule discovered is ascorbic acid, i.e., vitamin C, that is, produced during aerobic metabolism, and reacts rapidly with O2^•-^, singlet oxygen and ozone (chemically), and H_2_O_2_ (enzymatically) through ascorbate peroxidase to neutralize their toxic effects. Besides this, in plants, such acid is also involved in the regeneration of carotenoids and vitamin E (tocopherol). The latter can also act as antioxidant and important liposoluble redox system, providing protection against lipid peroxidation [24]. Data from* in vitro* studies have shown that *β*-carotene can regenerate tocopherol from the tocopheroxyl radical; then the resulting carotenoid radical cation may be repaired by vitamin C [25]. The role of vitamins as potent antioxidants has been recently reviewed [26]; therefore, we decided to be more focused on other food-derived phytochemicals.

Carotenoids, responsible for many of the red, orange, and yellow hues of flowers, leaves, and fruits, are powerful antioxidants. Readily available due to their large occurrence in fruits and vegetables (Table 1), their antioxidant activity is based on the scavenging peroxyl radicals [27]. The number of conjugated double bonds of these molecules is related to the efficiency of quenching, i.e., *α*- and *β*-carotene, but also zeaxanthin, cryptoxanthin belong to the group of highly active quenchers of ^1^O_2_ [25]. For example, lycopene, an intermediate in biosynthetic pathway of carotenoids, acts as scavenger of ROS, lipid peroxyl radicals, and nitric oxide and may exert protective activity against cancer, atherosclerosis, diabetes, and diseases related to inflammatory processes [28]. Synergistic effects in scavenging reactive nitrogen species have been reported for *β*-carotene and vitamins C and E [25]. While several data show the antioxidant activity of carotenoids, controversy exists about their antioxidant potential in biological systems, since a number of factors may affect their efficacy and, in some cases, prooxidant effects at high concentration and oxygen pressure have been reported [29].

The most promising molecules for further health promoting studies are phenolic compounds. These phytochemicals comprehend a vast range of molecules (about 8000 different structures), playing important roles in the life of plants [30], where they are widely distributed. These compounds can be divided into phenolic acids, lignans, lignins, stilbenes, tannins, and flavonoids, as schematically summarized in Figure 1. Even though they are constitutively present, stressful growth conditions and/or changes of growth medium components may further induce their synthesis and can be used to enhance their production by* in vitro* plant cultures [31, 32]. In plants, phenolics are involved in H_2_O_2_ detoxification, providing protection against UV radiation, also acting as enzyme modulators and feeding deterrents for herbivores [33]. The broad spectrum of biological activities of phenolics, among which antioxidant (i.e., reducing agents, free radical scavenger, and quenchers of single oxygen formation) and antitumor properties, is widely acknowledged in several studies [23, 34]. The presence of at least one phenol ring is important for such activity, with hydroxyl, methyl, or acetyl groups replacing the hydrogen. An increased antioxidant activity has been related to the enhanced number of free hydroxyls and conjugation of side chains to aromatic rings [35].

Flavonoids contain the following subclasses: flavonols, flavones, flavanones, flavan-3-ols, isoflavones, and anthocyanidins. They have attracted the attention of the researchers because of their positive effects on a number of diseases as reported in this review. For instance, quercetin and anthocyanins have been reported to be effective in reducing the growth rate of malignant cells, influencing carcinogen metabolism, reducing parameters of tissue inflammation, and inhibiting angiogenesis [7, 36]. According to some authors, the antitumor activities of phenolic compounds may be related to apoptosis, scavenging of radicals, antioxidant, and prooxidant characteristics [37].

Terpenoids represent another very large family of plant secondary metabolites [38].* In vitro* assays showed that monoterpenes, sesquiterpenes, and diterpenes extracted from aromatic plants have notable antioxidant activity [39]. The hypoglycemic and antioxidant activity of the alkaloid vindoline, vindolidine, vindolicine, and vindolinine, obtained from* Catharanthus roseus* leaves, have been reported [40]. Moreover, vindolicine shows the highest antioxidant effects, and also decreases H_2_O_2_-induced oxidative damage to pancreatic cells, suggesting it as a potential antidiabetic agent.

## 3. Natural Compounds and Inflammation

It is well known that ROS represent physiologic activators of transcription factors, such as Activator Protein-1 (AP-1) or Nuclear Factor *κ*B (NF-*κ*B), which in turn are able to modulate the transcription of proinflammatory cytokines such as Tumor Necrosis Factor *α* (TNF-*α*), Interleukin (IL)-6, IL-8, and IL-1 [41]. In fact, ROS, acting as an intracellular signaling component, are associated with inflammatory response and with autoimmune diseases [42]. Therefore, the use of natural products with antioxidant and anti-inflammatory activity represents an intriguing strategy for future clinical applications. These natural compounds have been shown to interfere with various proinflammatory mediators. Herbal medicines, nutraceuticals, which contain food or plant-derived constituents, or functional foods with anti-inflammatory features, can be used as complementary to anti-inflammatory drugs leading to the reduction in dose level of such drugs, thereby reducing their side effects. Phytochemicals with anti-inflammatory activities have been systematically reviewed [43].

A wide range of flavonoids with various chemical structures was associated with different anti-inflammatory mechanistic effects [44]. Glycosides of apigenin and luteolin are the most diffuse flavones. Important edible sources of flavones are parsley, rosemary, and celery [45]. Apigenin suppresses nitric oxide (NO) and prostaglandin production via inhibition of inducible nitric oxide synthase (iNOS) and COX-2, respectively [46]. Luteolin was also proved to inhibit chronic inflammation by* in vitro* co-culture of adipocytes and macrophages and the phosphorylation of JNK in macrophages [47]. One of the most studied flavonols is quercetin, which may be found in various vegetables and fruits, such as apples. Apple flavonoids have been associated with anti-inflammatory effects. In particular, quercetin and its glycosides were demonstrated to be potent anti-inflammatory agents on sarcoidosis patients and* in vivo* models of arthritis and allergic airway inflammation [48, 49].

Flavonoids and flavones from fruits of the* Citrus *spp. inhibit a range of pro-inflammatory mediators, including those derived from the arachidonic acid cascade [50]. In fact,* Citrus *flavanones (e.g., naringenin) mediate anti-inflammatory effects by modulating neuro-inflammation via interaction with p38 signaling cascades and STAT-1 [51] or suppressing the inflammatory response in an animal model of arthritis when administered orally [52].

Genistein, daidzein, and glycitein are isoflavones found almost exclusively in leguminous plants like soya (*Glycine max*). Genistein may inhibit inflammation inhibiting NF-*κ*B activation, downregulating TNF-*α* and IL-6 expression and secretion, endothelin-1, and vascular cell adhesion molecule-1 (VCAM-1) expression [53]. In plants, flavanols can occur as monomers (e.g., catechin and epicatechin) and oligomers or polymers, described as proanthocyanidins or condensed tannins. Catechins interfere with the inflammatory processes that contribute to atherosclerosis progression [54], while, among the effects of flavan-3-ols, present in dietary plants, like tea (*Camellia sinensis*) and cocoa (*Theobroma cacao*), inhibition of eicosanoid production, reduction of platelet activation and modulation of NO-dependent mechanisms, and modulation of proinflamamtory cytokine production can be included [55]. Anthocyanins exert their anti-inflammatory effects, particularly via the mitogen-activated protein kinase (MAPK) pathway [56]. Mechanistic studies report the glycosides of malvidin, delphinidin, cyanidin, petunidin, and peonidin to dose-dependently reduce IL-1*β* -activation of NF-*κ* B* in vitro* [57].

Diterpenes from* Stevia rebaudiana* leaves, used as a source of natural sweeteners in the food industry, have also been shown to attenuate proinflammatory cytokines (TNF-*α*, IL-1*β*, and IL-6) production via modulation of the I*κ*-B*α* /NF-*κ*B pathway [58]. Triterpenes from licorice root (*Glycyrrhiza glabra*) are glycyrrhizin and glycyrrhetinic acid: they have several effects, including gastric protection and modulation of blood pressure through their mineralocorticoid activity [59], but also anti-inflammatory function acting* via* the P13K/Akt/GSK3-*β* pathway to reduce cytokine production, while 18 *β*-glycyrrhetinic acid also blocks inflammation by causing dissociation of the glucocorticoid receptor [60].

Curcumin was demonstrated to inhibit LPS-induced production of TNF-*α* and IL-1*β* in a human monocytic macrophage cell line and, at the same millimolar concentration, to inhibit LPS-induced activation of NF-*κ*B and reduce the biological activity of TNF-*α* [61].

Stilbenes are found in only low quantities in the human diet. Resveratrol inhibits TNF-*α*, IL-1*β*, and IL-6 expression [62] and its ability to suppress NF-*κ*B activation, possibly via SIRT-1 activation, is suggested to be important in counteracting microglial inflammation. Rosmarinic acid is a phenolic acid occurring in herbs such as rosemary and sage. Its anti-inflammatory effects were shown by testing its topical application improving symptoms in atopic dermatitis patients [63].

Chronic inflammation is the main pathogenetic factor of many autoimmune diseases whose treatment is based on long or life-long administration of anti-inflammatory drugs. The possibility to use safe and effective natural products to reduce the dosage and side effects of conventional drugs represents an interesting field of study. Monoclonal antibodies against TNF-*α* or TNF-*α* soluble receptors are among the most efficient biological DMARDs (disease-modifying antirheumatic drugs) available for the chronic treatment of rheumatoid arthritis (RA) and other chronic inflammatory diseases. In nature, many natural compounds have been found to reduce the levels of TNF-*α* by interfering with various pro-inflammatory mediators and upstream targets. Thus, these compounds may represent alternative or adjunctive means of treating inflammatory diseases by modulating production, rather than activity, of TNF-*α* (reviewed in [64]).

On the other hand, fatty acids and their derivatives were shown to exert profound suppressive effects on the expression of iNOS, COX-II, IL-6, and TNF-*α* via a blockade of the NF-kB and AP-1 pathways. The strong anti-inflammatory potential and improved clinical parameters of RA of marine n-3 long-chain PUFA were also reported by Barden et al. [65].

Among flavonoids, luteolin and quercetin were the most potent TNF-*α* inhibitors [66]. The anti-inflammatory power of quercetin was clinically tested in women with RA demonstrating a significant effect in controlling inflammation and clinical symptoms [67]. Irrespective of their activity on the synthesis of TNF-*α*, green tea extract has also been proven in recent clinical trials to have anti-inflammatory and immunomodulatory properties in autoimmune disease [68].

Therefore, although natural product probably cannot substitute anti-inflammatory drugs, including DMARDs, they could significantly contribute to the reduction of dosage, for a more economic and safer treatment of autoimmune, as well as many other inflammation-related diseases.

## 4. Oxidative Stress, Metabolic Syndrome and Aging

Aging is a series of morphological and functional changes taking place over the time that can lead to deterioration of the biological functions of an organism [69]. ROS generated as byproducts of biological oxidations can induce severe oxidative damage to macromolecules eventually leading to cellular dysfunction [70]. Combination of aging, insulin resistance, and CVD can precipitate into metabolic syndrome [69, 71]. Although insulin resistance is considered as the main pathogenic mechanism-underlying onset of metabolic syndrome, a low-level chronic inflammation and oxidative stress have received much attention recently [72]. Additionally, evidences from experimental and clinical studies have shown that oxidative stress is a pivotal factor for obesity-associated diabetes, metabolic syndrome, and nonalcoholic steatohepatitis (NASH) [73, 74]. On the other hand, metabolic syndrome is the major health challenge of the 21^st^ century that can significantly affect ever-increasing life and health spans in the developed world. Although the exact mechanism responsible is largely unknown, it is considered that metabolic syndrome can significantly advance aging.

Since the ancient times, natural products have always been suggested to improve longevity of an organism [75]. Epidemiological and experimental data suggest natural products to be powerful antioxidants that can improve stress-related diseases. Scientific literature also suggests potential of natural products in improving metabolic syndrome and aging [76, 77]. Therefore, detailed preclinical evaluation on underlying mechanism of action and bioactivities of the natural compounds may provide solid scientific foundation for clinical applications.

Polyphenols and in particular flavonoids have been shown to protect from various age-related disease [78]. Several studies have indicated that supplementation with dietary polyphenols such as (−)-epigallocatechin-3-gallate (EGCG) and curcumin can improve age-associated cellular damage by reducing generation of ROS [79]. On the other hand, resveratrol and pterostilbene are considered excellent as anti-aging natural compounds that can modulate oxidative damage, inflammation, and cell senescence; components associated with aging [80] as well as flavonoids have been shown to improve aging mainly by controlling metabolic syndrome [81]. Some of the commonly reported flavonoids that can tackle one or more components associated with aging or metabolic syndrome are hesperidin, hesperetin, naringin, and naringenin [82].

It is noteworthly that the literature on preclinical use of phytochemicals to treat various conditions associated with aging is ever expanding. However, a large number of phytochemicals have been tested successfully in clinical trials for age-related condition. It is noteworthy that some limitations of pre-clinical studies that can affect their translational significance are (1) choice of experimental models that is not clinically relevant, (2) poorly characterized mechanism of action, and (3) clinically irrelevant dosing/time points for data interpretation.

## 5. Natural Compounds and Vascular Diseases

Alteration of vascular function is a key pathogenic process common to many important and highly diffuse human pathologies [83]. The morpho-functional integrity of the vascular endothelium is a complex and highly homeostatic process involving maintenance of vasorelaxation ability as well as anti-inflammatory and barrier functions with important effects on atherogenesis and increased risk of cardiovascular diseases (CVD) [84, 85].

Aging and chronic inflammatory conditions, such as diabetes, alter vascular homeostasis disrupting the “protective” functions of the vascular endothelium, a mechanism known as vascular dysfunction [86]. Physiological aging progressively deteriorates vascular function; however, poor life style, hyperlipidemia, and hyperglycemia associated with oxidative stress can significantly accelerate these pathologic processes leading to CVD and macro- and microvascular complications of diabetes mellitus [87], including ocular pathologies such as ischemic retinopathies. Crucial to the development of vascular dysfunction in aging is the induction of oxidative/nitrative stress [88], which is also involved in the pathogenesis of other human diseases discussed in this review.

Vascular redox imbalance linked to aging and diabetes share important common features such as the induction of the so-called senescence-associated secretory phenotype (SASP) [89]. According to this emerging concept, aging and metabolic stress lead to redox imbalance and trigger enhanced expression of senescence markers and production/secretion of inflammatory cytokines [90, 91]. Suppression of sirtuins, class III NAD^+^-dependent protein histone deacetylases, is an important feature of SASP [92]. In particular, SIRT-1 plays a key role in maintenance of these cellular homeostasis and energy metabolism [93, 94]. The role of natural compounds as potent anti-oxidant players able to prevent, at least partially, pathologic processes associated with aging and metabolic diseases was recently reviewed [95]. Here we will focus on the therapeutic effects of omega 3 poly-unsaturated fatty acids (PUFA) and the flavonoid resveratrol because of their predominant effects on vascular dysfunction, SASP and CVD.

Omega 3 poly-unsaturated fatty acids (PUFAs) include alpha linoleic acid (18:3) (ALA), eicosapentaenoic acid (EPA) (20:5n-3), and docosaexhaenoic acid (DHA) (22-6n-3). ALA is not synthesized in humans and is considered plant-derived omega 3, whereas EPA and DHA are found predominantly in fish. The effects of omega 3 PUFAs are primarily attributed to their lipid lowering effects and consequent reduction of risk of atherosclerosis [96]. Omega 3 PUFAs have shown to reduce vascular inflammation by downregulating adhesion molecules and limiting leukocytes adhesion to the vascular wall [97]. This latter directly influences production of endothelial-derived nitric oxide due to stabilization of lipid rafts such as the endothelial cells caveolae, as demonstrated in retinal endothelial cells [98]. However, the experimental studies appear to be much more supportive of PUFA's positive effects than the clinical evidence. In fact, the omega 3 PUFA effects on increased endothelial regenerative capacity and maintenance of vascular endothelial cells homeostasis due to membrane stabilizing ability were demonstrated to haveimportant effects on prevention od CVD [98]. An extensive review of the literature, recently appeared on Cochrane Database Systematic Review [99] and summarized the results of a large number of randomized clinical trials assessing the effects of different doses of PUFA on CVD outcomes. The results of this study showed that higher PUFA intake only slightly reduces risk of coronary heart disease and CVD acute events (i.e., stroke) and mortality, but overall has not significant effects on all-cause or cardiovascular disease mortality. Most of the positive effects were associated with modulation of lipid metabolism [99]. In any case, even a slight but significant reduction of 10% of morbidity and mortality for CVD associated with PUFA supplementation remains a significant clinical outcome [100].

The cardioprotective effects of resveratrol in human studies have been reported [101]. However, the* in vivo* evidence and the clinical studies are less conclusive, mostly because of the poor intestinal absorption of these flavonoids and the extensive degradation in various phenolic acids, which may retain some antioxidant activity [102]. It is noteworthy that one additional function of resveratrol was linked to enhanced SIRT-1 expression and activity and was extensively linked to longevity [103], while a prooxidant activity of resveratrol under certain conditions was reported [104].

Ultimately, the deleterious effects of aging and metabolic diseases (i.e., diabetes) in promoting oxidative stress and vascular dysfunction can negatively affect the cardiovascular system by promoting vascular dysfunction. There is no doubt that a correct life style including a balanced healthy nutrition, physical exercise, and the use of nutraceuticals can positively impact longevity by preventing CVD. However, when CVD pathologies have been established, nutraceuticals such as omega 3 and resveratrol can still find therapeutic application as effective adjuvant therapies because of their numerous positive effects on vascular homeostasis.

Finally, other phytochemicals with antioxidant effects have been reported to play a protective action on the risk or the development of CVD and therefore were proposed as important factors in diet, like, for instance, *β*-carotene, curcuma, and others [105, 106]. In particular, several studies underline the anti-atherogenic effect of lycopene in association with the inhibition of proinflammatory cytokines secretion [107].

## 6. Phytochemicals for Neurodegenerative Diseases Prevention

Neurodegenerative diseases (NDDs) are a heterogeneous group of chronic and untreatable conditions, characterized by progressive functional impairment of the nervous system, induced by deterioration of neurons, myelin sheath, neurotransmission, and movement control. Among one of the most disabling of these, Alzheimer's disease (AD) is a NDD that destroys memory and other important mental functions. AD is characterized by the accumulation of amyloid-beta peptide (A*β*) in the brain, the presence of neurofibrillary tangles (NFTs) containing hyperphosphorylated tau fragments, and the loss of cortical neurons and synapses [108]. Parkinson's disease (PD) affects predominately dopaminergic neurons in the substantia nigra, associated with accumulation of Lewy bodies containing *α*-synuclein in neurons and increased neuroinflammatory cells. Huntington disease (HD) is a progressive brain disorder that causes uncontrolled movements, emotional problems, and loss of thinking ability and occurs in early middle life, even if it is recognized as a juvenile form. HD is an autosomal dominant NDD, characterized by the abnormal expansion of the CAG triplet repeats in the polyglutamine region of the huntingtin (HTT) gene [109]. Multiple sclerosis (MS) is a chronic, autoimmune, inflammatory disease that affects the brain and spinal cord, caused by autoimmune-mediated loss of myelin and axonal damage [110]. At present, there is no effective treatment for NDDs, and, in order to identify novel therapy or adjuvant strategy for NDDs, several natural medicinal plants have gained attention as potential neuroprotective agents, and a number of studies have suggested that a diet rich in vegetable products can prevent or delay the NDDs onset [111]. These properties might be due to the presence of polyphenols, an important group of phytochemicals that are abundantly present in fruits, vegetables, cereals, and beverages (Table 1) and already discussed in other sections of this review. In this chapter, we focused on the potential role of polyphenols for preventive and therapeutic purposes for NDDs treatment, based on related research evidence.

Resveratrol demonstrates significant neuroprotective activity both* in vitro* and* in vivo*. Several studies have demonstrated that resveratrol is cytoprotective in cells exposed to A*β* and/or to A*β*-metal complex via Sirt3-mediated mechanisms [112, 113].* In vivo*, in a mouse model of cerebral amyloid deposition, orally administered resveratrol lowered microglial activation associated with cortical amyloid plaque formation [114]. Furthermore, long-term dietary resveratrol reduces cognitive impairment and has a neuroprotective role, decreasing the amyloid burden and reducing tau hyperphosphorylation in SAMP8 mice, a model of age-related AD [115]. Increasing evidence has also suggested that resveratrol shows enhanced benefits in cell and animal models of PD. In rat primary midbrain neuron-glia cultures, resveratrol protected dopaminergic neurons against lipopolysaccharide (LPS)-induced neurotoxicity in concentration- and time-dependent manners, through the inhibition of microglial activation and the subsequent reduction of proinflammatory factors release [116].* In vivo*, resveratrol ameliorated both motor deficits and pathological changes in MPTP-treated mice* via *activation of SIRT-1 and subsequent LC3 deacetylation-mediated autophagic degradation of *α*-synuclein [117]. All the above findings suggest that resveratrol may be a potential prophylactic or therapeutic agent for NDDs, with the caution reported elsewhere in this review regarding intestinal absorption.

Curcuminoids consist of three components: curcumin (75%–80%), demethoxycurcumin (15%–20%), and bisdemethoxycurcumin (3%–5%). Curcumin also induces neuroprotective effects through the control of pathogenetic oxidative and inflammatory mechanisms both* in vitro* and* in vivo* models of AD and PD. In Neuro2a mouse neuroblastoma cells infected with Japanese encephalitis virus, curcumin enhances cell viability by decreasing ROS and inhibiting proapoptotic signals [118]. Furthermore, curcumin protects against *α*-synuclein-induced cytotoxicity in SH-SY5Y neuroblastoma cells decreasing cytotoxicity of aggregated *α*-synuclein, reducing intracellular ROS, and inhibiting caspase-3 activation [119].* In vivo*, curcumin significantly alleviated spatial memory deficits in APP/PS1 mouse model of AD, promoting cholinergic neuronal function [120]. Curcumin also reduced the activation of microglia and astrocytes, as well as cytokine production and inhibited NF-*κ*B signaling pathway, suggesting the beneficial effects of curcumin on AD mice are attributable to the suppression of neuroinflammation [120]. In the PD animal model induced by the neurotoxin MPTP, curcumin is neuroprotective and prevents glutathione depletion and lipid peroxidation induced by this toxin. More recently, curcumin restored motor deficits and enhanced the activities of antioxidant enzymes in rotenone induced Parkinson's in mice [121]. All these findings suggest a neuroprotective role of curcumin, and offer strong justification for the therapeutic prospective of this compound in the management of NDDs.

Pretreatment of primary hippocampal cultures with quercetin significantly attenuated A*β*(1-42)-induced cytotoxicity, protein oxidation, lipid peroxidation, and apoptosis by modulating oxidative stress [122]. More interestingly, quercetin decreases extracellular *β*-amyloidosis, tauopathy, astrogliosis, and microgliosis in the hippocampus and the amygdala and improves performance on learning and spatial memory tasks in aged triple transgenic Alzheimer's disease model mice [123].

Taken together, the above evidences suggest polyphenols as neuroprotective agents. The habitual consumption of dietary polyphenols is proven to inhibit various secondary sources of ROS and proinflammatory cytokines, thus reducing the risk of NDDs [124]. A beneficial clinical use of polyphenols to attenuate oxidative damage in aging and age-related disorders may be a viable and promising approach for the prevention and treatment of NDDs.

## 7. Natural Compounds as Anti-Cancer Agents 

Although large progress was achieved, some tumors still present poor prognosis and research is currently geared towards the use of non-toxic doses of plant-extracted compounds. The road for a new therapeutic approach, based on natural molecules and drugs, was opened by the identification and use of natural chemotherapeutic agents like taxanes, vinca alkaloids, and anthracyclines [125]. Therefore, it is logical to hypothesize that compounds found in foods are likely to have some protective effects.

**Figure 3:**
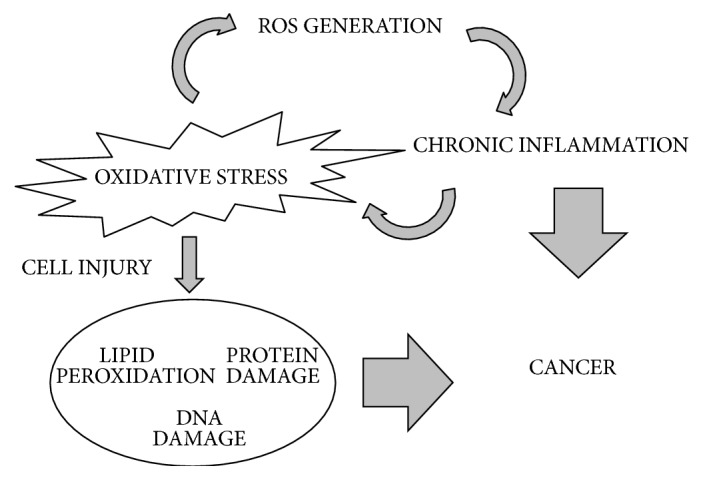
Schematic view of complex cross-talk between oxidative stress, chronic inflammation, and cancer.

Numerous studies suggest that chronic inflammation is able to promote all stages of cancer development, including initiation, progression, and metastatic potential [126]. Moreover, recent data [127] show a close relationship between age-related pathologies, including cancer, and inflammation (Figure 3). ROS are mainly responsible of inflammation and cancer promotion by oxidative stress [128]. In particular, there are increasing evidences on the role of TNF-*α*, a well-known pro-inflammatory cytokine and a regulator of the generation of ROS, in the promotion of carcinogenesis through the activation of the transcription factor NF-*κ*B [129].

Interestingly, recent literature data indicate an emerging role of polyamines metabolism as a novel target against inflammatory diseases. Polyamines are naturally occurring aliphatic compounds, ubiquitous to all living organisms, which interact with DNA, RNA and proteins and are required for eukaryotic cell growth, survival, and differentiation. Notably, excessive polyamine catabolism can lead to ROS formation, increasing oxidative stress, with subsequent enhancement of inflammatory response [130]. For that reason, polyamines metabolism represents an interesting target for anticancer therapy using natural compounds [131]. Therefore, the use of different natural substances, mainly polyphenols, coming from plants and foods, may exert promising results in antitumor therapy due to their anti-oxidant activities [132]. In particular, diet with high polyphenol content has been shown positive effects against cancer-related anorexia/cachexia syndrome and oxidative stress [133].

The flavonol quercetin is a well-known antioxidant molecule with well-documented anticancer activity [134]. Mechanisms underlying its anticancer activity are not completely elucidated; however, it is known that quercetin affects negatively the synthesis of polyamines, well known growth factors, by the inhibition of ornithine decarboxylase (ODC) expression [135]. Interestingly, it has been demonstrated that high flavonols intake, especially quercetin and kaempferol, is able to induce a significative reduction of serum IL-6 concentration, a well-known inflammation-reletated cytokine [136].

Also curcumin shows anti-inflammatory and anti-oxidant properties and potential anti-cancer activity [137]. Indeed, this molecule has been tested in a wide range of cancer cell lines, like cervical cancer [138], colorectal cancer [139], and breast cancer [140]. Moreover, curcumin has been shown to have effects on many signalling and polyamine pathways [141]. Although several studies underline curcumin therapeutic efficacy, its clinical administration is difficult due to its poor oral bioavailability, low solubility, and degradation [142]).

Resveratrol shows a potent cytotoxic effect on cancer cells [143]. For example, recently it was reported that this effect is abolished by Transglutaminase type 2 inhibition on cholangiocarcinoma and gallbladder cancer cell lines [144]. In the last decades, resveratrol has been one of the most studied natural compounds, often leading to contradictory results [145]. Although resveratrol is considered a good candidate as chemopreventive and synergistic agent, further studies are needed [146].

Genistein is a soybean isoflavone. The antitumor activity of genistein has been observed in various forms of cancer such as neuroblastoma and chronic lymphatic leukemia and in several organs such as breast, ovary, prostate, urinary bladder, colon, liver, and stomach [147]. Due to its structural similarity to mammalian estradiol, genistein is known as a phytoestrogen. A large number of studies suggest a beneficial role of this isoflavone in the inhibition of carcinogenesis in animal models. Although the effects of genistein as chemoprevention agent remain controversial [148], several human intervention studies have been undertaken. In fact, it has been demonstrated that dietary soy supplementation may reduce inflammatory processes related to prostate carcinogenesis [149].

One of the most well-known anticancer agents with anti-oxidant and anti-inflammatory properties is lycopene that exerts therapeutic effects on a large variety of cancers [150, 151]. In particular, it has been demonstrated that lycopene intake reduces prostate cancer risk [152].

During the last few decades, it has emerged how genomic instability, telomere attrition and epigenetic changes may underlie aging and senescence phenomena [153]. Telomeres are short tandem repeated sequences (TTAGGG) that are localized on the 5' ends of chromosomes. The length of telomeres is guaranteed by the activity of telomerase enzyme, widely expressed in tumor cells. The G-rich telomeric sequence can assume G-quadruplex DNA secondary structures, able to inhibit telomerase activity. Therefore, inhibition of telomerase or the stabilization of G-quadruplex by natural compounds may represent an important anti-cancer strategy [154, 155].

The identification of natural compounds has contributed to the improvement of cancer therapies and indeed many of these molecules are currently used in clinical practice. It is important, however, to emphasize that rigorous studies and preclinical investigations are needed to clarify their potential chemopreventive and antitumor activities.

## 8. Phytochemicals in Skin Diseases

According to the European Medicines agency (http://www.ema.europa.eu/ema/), several studies analyze in depth the actual efficacy of herbal medicinal products and derived molecules. Herbal products can be sorted according to their clinical use in several diseases such as appetite disorders, sleep disorders, pain and inflammation control, eye disorders, gastrointestinal disorders, and others (http://www.ema.europa.eu/ema/index.jsp?curl=pages/medicines/landing/herbal_search.jsp&mid=WC0b01ac058001fa1d for a complete list). We focus here the attention on the beneficial use of herbal medicinal products in skin disorders, with a specific attention on pathologies often related to the skin-aging process [156]. Since in most cases they have been studied or proposed through topical applications, they are often referred to as oils or herbal preparations whose classification in terms of phytochemical content is rather complex.

Therapeutic applications to skin pathologies are proposed for Agrimoniae herba (*Agrimonia eupatoria*; in minor inflammation and superficial wounds),* Echinacea purpurea* (in small superficial wounds and mild acne), Soiae oleum (*Glycine max*; in mild recurrent eczema), Juglandis folium (*Juglans regia*; in minor skin inflammation), Matricariae aetheroleum (in anous and genitals irritation), Matricariae flos (*Matricaria recrutita*; in mild skin inflammation and sunburns and superficial wounds),* Melaleuca* spp. (in insects bites, mild acne, itching, minor skin inflammation), Meliloti herba (*Melitotus officinalis*; in minor skin inflammation), Origani dictamni herba and Origani majoranae herba (*Origanum* spp.; in minor skin inflammation and irritation), Rosae flos (*Rosa* spp.; in skin and mouth inflammation), and Solani dulcamarae stipites (*Solanum dulcamara*; in itchy and rash from mild eczema) (see http://www.ema.europa.eu/ema/index.jsp?currentCategory=Skin+disorders+%26+minor+wounds&curl=pages%2Fmedicines%2Flanding%2Fherbal_search.jsp&mid=WC0b01ac058001fa1d&searchType=Latin+name+of+herbal+substance&taxonomyPath=&keyword=Enter+keywords&alreadyLoaded=true&treeNumber=&searchkwByEnter=false&pageNo=2 for more details).

The European Medicines Agency official list of herbal substances, preparations, and combinations for use as traditional herbal medicinal products contains 12 substances, according to the European Community decision reported at the EU site (https://eur-lex.europa.eu/legal-content/EN/TXT/?uri=CELEX:02008D0911-20180126).

The 12 herbal substances are as follows:* Calendula officinalis, Echinacea purpurea, Eleutherococcus senticosus, Foeniculum vulgare, Hamamelis virginiana, Melaleuca spp., Mentha spp., Pimpinella anisum, Sideritis scardica, Thymus vulgaris, Valeriana officinalis*, and* Vitis vinifera*.

Their introduction within such official list is sustained by scientific reports demonstrating their therapeutic effectiveness in different pathological settings. We collected the scientific literature reported in PubMed for each of these substances co-occurring in the ALL fields with any of the common skin diseases reported in the following list: acne, allergy, basal cell carcinoma, blister, carbuncle, cellulitis, chickenpox, dermatitis, eczema, hives, impetigo, lupus, measles, melanoma, psoriasis, ringworm, rosacea, squamous cell carcinoma, vitiligo, and wart.

**Figure 4:**
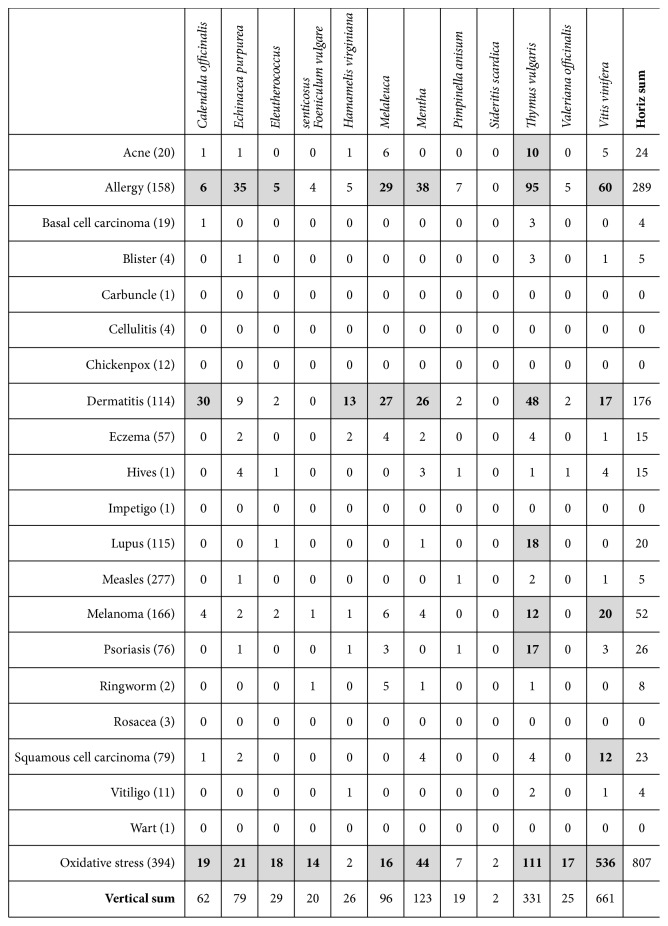
Number of published studies regarding plant-derived nutraceuticals and age-related skin diseases. In the first column, within brackets, the number of PubMed abstracts containing in the Title field the diseases name and the word “age” is reported. In several diseases a strong relation with the age is evident (namely, in allergy, basal cell carcinoma, chickenpox, dermatitis, eczema, lupus, measles, melanoma, psoriasis, squamous cell carcinoma, and oxidative stress). In the other columns, the number of PubMed abstracts containing the diseases name and the phytochemical name in All field is reported. Co-occurrence > 10 is highlighted in bold and gray background. The raw named Oxidative stress reports the number of studies indexed on PubMed containing experimental data which correlate each plant-derived nutraceutical with an “oxidative stress” (as present in All field of database).

As reported in Figure 4, the investigated phytotherapeutic agents are mostly associated to allergy, dermatitis, and melanoma, related in most cases to either* Melaleuca*, or* Mentha* or* Thymus vulgaris *or* Vitis vinifera*.

More in detail, local application of* Melaleuca alternifolia* derived oils has been consistently reported to achieve a significant improvement of acne lesions, according to several independent studies [157]. The clinical efficacy of Melaleluca oil is likely related to its known antibacterial action [158] as well as anti-inflammatory activity [159]. The essential oil from* Melaleuca alternifolia* also shows antioxidant activities potentially useful in dermatitis and skin cancers [160]. Extracts from* Melaleuca quinquenervia* have been shown to inhibit melanin content in mouse melanoma cells, thus exerting potential cosmetic applications [161]. Minor side effects are associated with* Melaleuca* such as burning, scaling, itch, redness, dryness, pruritus, and stinging. Contact allergy to Melaleuca oil has been reported in some cases as well as dermatitis reactions indicating the essential oil from* Melaleuca* as a sensitizers and potentially irritant agent. Mentha-derived oil is used to relief skin inflammation and pruritus [162]; nevertheless, allergic reactions have been reported in some cases.

Finally, regarding* Vitis vinifera*, a meta-analysis recently published demonstrates* Vitis vinifera* as one of the effective components of medical devices useful in atopic dermatitis local treatment [163]. Interesting results have also been collected in oncological conditions;* Vitis vinifera* has shown some efficacy in reducing radiotherapy-induced dermatitis [164] and in inhibiting cell proliferation in melanoma [165, 166] and skin non-melanoma cancer [167], indicating grape seed proanthocyanidin as an apoptosis and autophagy inducer. Rare allergic reactions are reported for* Vitis vinifera* [168].

Hundreds of other phytochemicals are reported in literature with potential effects on skin, such as anti-age activity [169], photoprotection [170], wound healing [171], and anti-infection [172].

## 9. Conclusions

The body of evidence reported in this review demonstrates the large interest around phytochemicals and their potential use against oxidative stress-related human diseases, with a focus on those in which inflamed cells play a crucial and pivotal role on pathogenic mechanisms. Their potential use, in combination with drugs like for instance DMARDs, may be very useful to reduce side effects and be cost-effective. Further, socioeconomical issues are playing an even more important role on the rate of new drugs development. This is coupled to an increasing interest toward the repurposing and repositioning of old drugs or others largely used in traditional medicine. Therefore, it is expected that in the next future phytochemicals-based drugs will be object of a growing interest for inflammation and oxidative stress-related diseases.

## Figures and Tables

**Table 1:** Some of the plant metabolites possessing antioxidant and antitumor activities.

Secondary metabolites	Common dietary sources	References
Polyphenols	Fruit, vegetables, coffee, tea, and cereals	[5, 6]

Anthocyanins	Strawberries, black rice, berries, cherry etc.	[7, 8]

Flavones	Blueberries, blood orange juice	[9]

Flavonols	Cherries, chokeberry, elderberries, Goji berry (wolfberry)	[9]

Resveratrol	Purple wine, peanuts	[10, 11]

Theaflavins	Black tea	[8, 12]

Carotenoids	Carrots, tomatoes, pumpkins, peppers, among others	[6, 13]

Lycopene	Tomatoes, watermelon, red peppers, papaya, apricot, pink grapefruit	[14, 15]
